# The cardiac autonomic nervous system: A target for modulation of atrial fibrillation

**DOI:** 10.1002/clc.23190

**Published:** 2019-05-06

**Authors:** Mu Qin, Cong Zeng, Xu Liu

**Affiliations:** ^1^ Department of Cardiology, Shanghai Chest Hospital Shanghai Jiaotong University Shanghai China

**Keywords:** atrial fibrillation, cardiac autonomic nerve system, neuromodulation

## Abstract

The cardiac autonomic nerve system (CANS) is a potentially potent modulator of the initiation and perpetuation of atrial fibrillation (AF). In this review, we focus on the relationship between the autonomic nervous system (ANS) and the pathophysiology of AF and the potential benefit and limitations of neuromodulation in the management of this arrhythmia from eight aspects. We conclude that Activation and Remodeling of CANS involved in the initiation and maintenance of AF. The network control mechanism, innervation regions, and sympathetic/parasympathetic balance play an important role in AF substrate. And the formation of Complex Fractional Atrial Electrograms also related to CANS activity. In addition, modulating CANS function by potential therapeutic applications include ganglionated plexus ablation, renal sympathetic denervation, and low‐level vagal nerve stimulation, may enable AF to be controlled. Although the role of the ANS has long been recognized, a better understanding of the complex interrelationships of the various components of the CANS will lead to improvement of treatments for this common arrhythmia.

## 1 INTRODUCTION

As early as 1682, William Harvey had described the close relationship between the nervous system and the heart.^1^ A large number of scholars have also demonstrated over the last half a century that cardiac autonomic nervous system (CANS) not only can regulate the heart rate and hemodynamic changes but also participates in the development of arrhythmia, especially atrial fibrillation (AF).^2^, ^3^, ^4^ Since the treatment of AF with antiarrhythmic drugs and radiofrequency catheter ablation has currently stuck in a bottleneck, exploring new pathogenic mechanisms will undoubtedly be a hot topic in the future research of AF theories. In particular, although the strategy of atrial autonomic ganglionated plexi (GP) ablation appears to be a “milestone” breakthrough in the field of AF prevention and treatment, a clinically effective GP modification criterion has not been detected, and present researches are facing a tight corner considering the high complexity of CANS mechanism and the unsatisfactory effect of clinical intervention. Based on the basic and clinical studies, we systematically reviewed the relationship between CANS and AF from different perspectives and elaborated our insight to the current status and potential problems of CANS research.

## 2 THE RELATIONSHIP BETWEEN FUNCTIONAL ALTERATION OF CANS AND AF

### 2.1 Activation and remodeling CANS in AF

**Figure 1:**
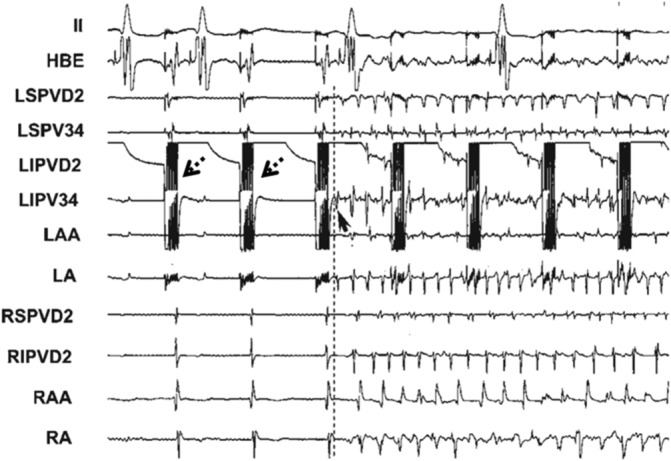
Atrial fibrillation (AF) was directly induced by high‐frequency stimulation (HFS) (2.4 V) at the pulmonary vein (PV). AV block was also observed before initiation of AF. HFS consisted of trains of high‐frequency electrical stimuli (200 Hz, 0.1 ms in duration of each impulse, train duration 40 ms, 0.6‐12 V), and were delivered at 2 ms after each pacing stimulus (two threshold at a cycle length of 330 ms)^6^

Coumel's triangle concept^5^ proposed that arrhythmogenic substrate, modulating factors, and triggering factors jointly produce arrhythmia. Apart from regulating the changes in cardiac rhythm, CANS has been shown to participate in the formation of AF pathogenic substrate and focal sources, thereby exerting an important role in AF triggering and maintenance. According to previous studies,^6^ stimulation of canine cervical vagal trunk with high‐frequency current will not only significantly shorten AF effective refractory period (ERP) but also result in increased incidence of AF, expanded window of vulnerability and prolonged AF duration. Some scholars have recently confirmed that high‐frequency stimulation (HFS) of atrial epicardial autonomic GP can cause an increase in ERP dispersion in multiple atrial sites and directly induce AF (Figure 1). However, this course can be eliminated by blocking vagus nerve function with drugs such as atropine. In addition, local application of acetylcholine (Ach) around the atrial GP tissue or in the left atrial appendage may also generate the similar atrial electrophysiological substrate changes and AF‐triggering effect as described above.^6^ Although the causal relationship between ANS and atrial arrhythmias still remains unclear because of the difficulty to directly record the nerve activity, Chen et al found that simultaneous sympathovagal discharges always preceded the onset of PAF in animal models. It implied that ANS activity may be an important trigger in the generation of PAF.^7^ These findings suggest that the activation of autonomic nerve can cause atrial electrophysiological changes through Ach released by nerve endings and further induce AF. Therefore, CANS may serve as the underlying manipulator of rapid atrial electrical activities.

Persistent or recurrent AF may lead to progressively shortened atrial ERP, increased ERP dispersion, reduced rate adaptability, and other atrial substrate remodeling changes, which is known as “AF begets AF” and considered to be an important mechanism for AF maintenance. In the rapid atrial pacing model (a classic model of “AF begets AF”), the 6‐hour rapid pacing (acute AF model) would induce atrial “electrical remodeling,” which is manifested by progressively shortened ERP and increased dispersion. Nevertheless, drug blockage of ANS by administration of autonomic blockers, atropine, and propranolol or GP ablation through the epicardium can decrease the heterogeneity across atria, thereby reversing and inhibiting the onset of atrial electrical remodeling.^8^ Continuous pacing for 4 to 6 weeks (persistent AF model) would increase the heterogeneity in the distribution and activity of atrial autonomic nerve, accompanied by variation in M receptor quantity and heterogeneity changes in the dynamic characteristics of *I*_K,Ach_ channel,^9^ which is known as “autonomic nerve remodeling”. Ng et al found that there was an increase in nerve bundle size, parasympathetic fibers/bundle, and density of sympathetic fibrils and cardiac ganglia in chronic heart failure canine model. β‐adrenergic blockade slowed AF dominant frequency, while parasympathetic blockade markedly decreased AF duration. It suggested that the ANS remodeling may contribute to AF substrate in CHF.^10^ Therefore, autonomic nerve activation/remodeling is not only associated with AF triggered activity but also participate in the maintenance mechanism of AF, that is, the development of atrial remodeling.

However, AF as a progressive disease contains three major components in human: initiation of the arrhythmia, arrhythmia maintenance, and progression toward longer‐lasting AF forms.^11^ The progression to sustained forms is estimated to be approximately 10% at 1 year and 25% to 30% at 5 years despite pharmacologic therapy. Multiple factors are involved in this progression, such as genetic predisposition, age, or disease‐related remodeling and the effect of “AF begets AF.” Therefore, we should recognize that these factors may make atrial remodeling more complicated in human AF than in rapid atrial pacing canine model.

### 2.2 The role of sympathetic and parasympathetic component of CANS in AF

**Figure 2:**
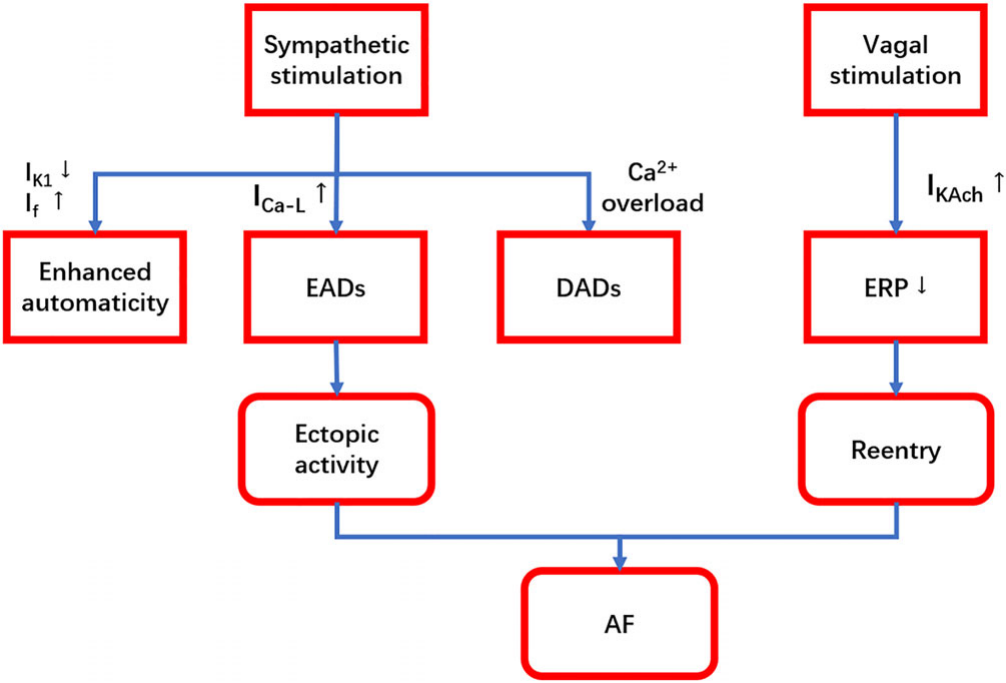
A simplified diagram interpreting the role of sympathetic and parasympathetic components of CANS in AF. Sympathetic stimulation leads to focal ectopic firing via enhanced automaticity, EADs and DADs. While vagal stimulation facilitates the maintenance of reentry by shortening the atrial ERP. And then AF can result from focal or re‐entrant mechanisms. AF, atrial fibrillation; CANS, cardiac autonomic nerve system; DADs, delayed afterdepolarizations; EADs, early afterdepolarizations; ERP, effective refractory period; up arrow: increase; down arrow: decrease

CANS can be divided into extrinsic CANS (ECANS) and intrinsic CANS (ICANS), where the former refers to brainstem and cardiac preganglionic fibers, while the latter consists of epicardial GP, fat pads, and related nerve fibers connecting them. ECANS comprises sympathetic nerves and vagus nerves, and the GP in ICANS also contain sympathetic components. They distinguish from each other by different activation effects, and the appropriate balance between them stabilizes and regulates the changes in heart rhythm. Vagal stimulation shortened the atrial ERP by augmenting *I*_K,Ach_ resulting in the decrease of the wave length for reentry, which made the maintenance of the reentry much easier.^12^ Po et al also demonstrated that in the presence of Ach, rapid, and sustained re‐entrant pulmonary vein (PV) tachycardias maintaining 1:1 conduction into LA at short cycle lengths can play a key role in the perpetuation of AF.^13^, ^14^ Furthermore, Lim et al reported that 91 ectopic activity was recorded in a total of 112 episodes of high‐frequency stimulation delivered at presumed GP sites to activate the ANS.^15^ It demonstrated that vagal excitation enhances a driving role of PV. While the activation of sympathetic nerves will promote ectopic activity through enhanced early afterdepolarizations (EADs) or delayed afterdepolarizations (DADs). Clinical evidence showed that the ligament of Marshall which was confirmed the presence of sympathetic nerve fibers and ganglion cells, can develop automatic rhythm during isoproterenol infusion and also contribute to the re‐entrant excitation in human AF.^16^ However, Tan et al found that the onset of AF was accompanied by the synchronous activation of sympathetic and vagus nerves and that the induction rate of AF under combined administration of isoprenaline and Ach was significantly increased compared with that under administration of Ach^17^ alone. This suggests that the synchronous activation of sympathetic and vagus nerves may serves as favorable contributing factor for AF onset (Figure 2).

In human, although many factors are involved in AF genesis, such as male sex, obesity, obstructive sleep apnea, alcohol consumption, endurance sports, and family history,^18^ clinical research has identified that most lone paroxysmal AF cases are vagus nerve‐dependent, whereas the sympathetic nerve‐dependent AF is often accompanied by organic heart diseases. In a study involving 30 patients with paroxysmal AF, Lo et al revealed that vagus nerve‐dependent AF was featured by increased left atrial myocardium voltage, shortened time for excitation and reduced atrial volume, while the sympathetic nerve‐dependent AF exhibited greater left atrial volume, more triggers outside the pulmonary vein and significantly elevated postoperative AF recurrence rate.^19^ In clinical studies, the classification of sympathetic nerve‐ and vagus nerve‐dependent AF is mainly based on the results of 24‐hour Holter HRV frequency domain analysis, where the case with more high‐frequency (HF) components and lower LF/HF ratio is defined as vagus nerve‐dependent AF, and vice versa for sympathetic nerve‐dependent AF.^20^ However, these parameters can only indirectly reflect autonomic nerve activity, and the direct evidence provided by human autonomic nerve activity record remains insufficient at present. In most cases, atrial rapid pacing, including intermittent pacing and continuous pacing, is employed for duplicating AF model in animal experiments.^21^, ^22^ Pacing frequency is set at 1000 bpm or faster with the pacing time of 7 to 14 days or longer. In this context, it is inevitable to affect heart functions and thus activate the sympathetic nerve; in addition, the activation of sympathetic or parasympathetic nervous system is always accompanied by the other. It is hard to quantify the activation intensity and interaction between the two components based on the current test technique. Therefore, effective scientific experimental methods and techniques are still lacking at present for exploring the effect of CANS sympathetic and vagal components in AF development. Although PET/CT has been widely applied,^23^ the detection of autonomic nerve activity requires further experience accumulation and technical support.

Based on the findings of previous studies, it can be presumed that sympathetic and vagus nerves play different roles in the regulation of atrial electrophysiological properties and they can both affect AF separately or function under a synergistic effect. However, either condition would break the original balance between sympathetic and vagus nerves. Recently, some scholars proposed the concept of “autonomic nervous imbalance” to explain the atrial electrical instability and AF onset caused by the stress of different autonomic nervous components. According to this concept, either the activation or prior activation of any CANS component can induce an imbalance between sympathetic and vagus nerves, and the key to achieve “rebalance” through interventions is to realize a quantitative evaluation on the activation intensity and activation time of different CANS components. However, this problem has not been reported in the existing literature and will undoubtedly be a new topic in the field of AF prevention and treatment in the future.

## 3 THE RELATIONSHIP BETWEEN REGULATED NETWORK OF CANS AND AF

### 3.1 The role of GPs connections in AF

**Figure 3:**
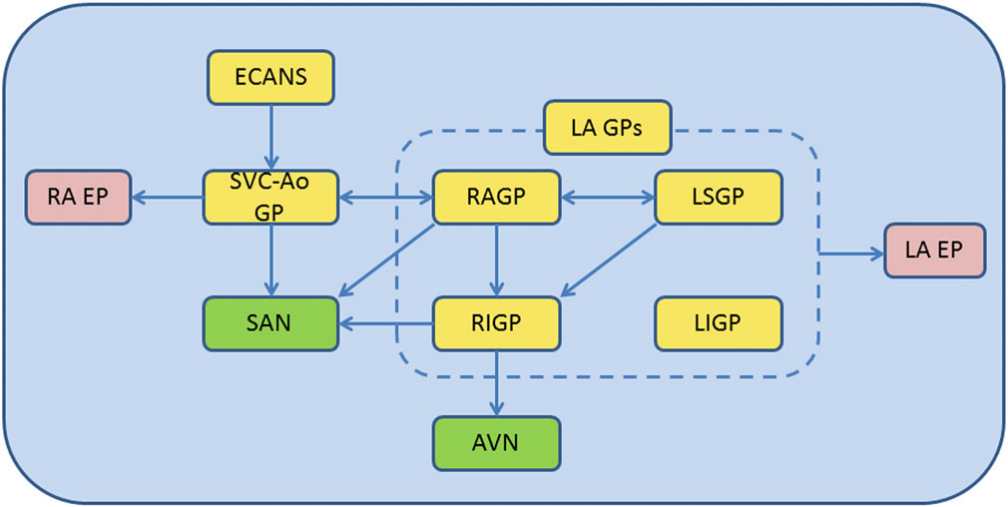
Summary of the interactions among extrinsic CANS (ECANS), superior vena cava‐aorta ganglionated plexus (SVC‐Ao GP), right anterior ganglionated plexus (RAGP), left superior ganglionated plexus (LSGP), right inferior ganglionated plexus (RIGP), left inferior ganglionated plexus (LIGP) on SA nodal (SAN), AV nodal (AVN) and atrial electrophysiology (EP). AVN, atrioventricular nodal; ECANS, extrinsic cardiac autonomic nerve system; LA EP, left atrial electrophysiology; LA GPs, left atrial ganglionated plexi; LIGP, left inferior ganglionated plexi; LSGP, left superior ganglionated plexi; RA EP, right atrial electrophysiology; RAGP, right anterior ganglionated plexi; RIGP, right inferior ganglionated plexi; SAN, sinoatrial nodal; SVC‐Ao GP, superior vena cava‐aorta

Apart from the regulatory action from ECANS, ICANS is also associated with the functional feedback regulation by its own complicated network as verified by extensive recent studies. Tan et al found in their canine AF model that 73% of AF or atrial tachycardia was accompanied by early ECANS activation^.^ 24 The study by Chen et al reported that ICANS activation was observed prior to the onset of paroxysmal AF in nearly 100% cases, where 20% suffered the attack in the absence of ECANS afferent signals,^25^ suggesting that ICANS could trigger AF completely independently of ECANS. The inherent complex network of CANS includes fat pads on the heart surface and GP surrounding the pulmonary vein, both of which act as key nodes in the autonomic nervous network control mechanism of AF. Many previous studies have revealed that any of GP would rapidly release acetylcholine and catecholamines under stimulation and further trigger AF. Then, is there an upstream‐downstream cross‐linking relationship between GP? Early studies have found that the third fat pad (SVC‐Ao GP) at the superior vena cava and aortic root delivers the nervous signal transmission between ECANS and ICANS as the “head station” of atrial GP afference. Lo recently proved that SVC‐Ao GP ablation could extend ERP and increase AF burden, which indicated that ECANS has an inhibitory effect on ICANS through SVC‐Ao GP.^26^ Meanwhile, the connection and regulation between GP within the ICANS network appears to be extremely complicated. Subject to the study by Moss et al, stimulation of left atrial GP would not only directly lead to changes in the left atrial ERP as well as Bachmann conduction bundle and sinuatrial node dysfunction but also affect the right atrial refractory period through directly or indirectly regulating the right atrial GP through SVC‐Ao GP.^27^ Based on the canine in vivo experiment, Hou et al found that right anterior GP (RAGP) might act as a relay station between atrial GP and exert a key regulatory effect on sinuatrial node, atrial refractory period, and AF; the left superior GP (SLGP) could regulate the function of sinuatrial node and atrioventricular node through the RAGP and right inferior GP (RIGP), respectively.^28^ In addition, our study also found that the Ao‐SVC GP is critical in modulating sinus nodal function through both RAGP‐dependent and ‐independent pathways.^29^ Based on the research results above, it is obvious that the cross‐linked structure of autonomic nervous network is highly complicated, that different GP are interconnected between each other and that the stimulation of single GP may cause multiple GP responses to produce a synergistic effect (Figure 3). In consequence, it is difficult to accurately locate the specific GP in the AF neurological regulation mechanism. We think that the concept of “CANS network control mechanism” provides a very appropriate explanation for the above‐mentioned phenomenon. That is, under direct stimulation or activation by nerve ending afferents, GP, as the network nodes, can cause a series or parallel signal transmission effect between GP and simultaneously generate positive feedbacks to amplify the above activation effect; as a result, the atrial myocardium will be exposed to the rapidly released massive neurotransmitters, which then leads to an acute change/remodeling in atrial electrophysiological properties, thus triggering and maintaining AF.

### 3.2 Innervation regions of CANS and AF

CANS is a highly integrated network, where the highly activated GP can release neurotransmitters by a proximal‐to‐distal gradient. The activation of axons from GP will then reversely activate the distant GP, thus leading to neurotransmitter release and inducing AF, which was explained by Sunny Po et al. with the “octopus hypothesis.”^30^ Recent studies indicate that multiple intra‐atrial regions are innervated by the nerve fibers from GP, called atrial innervation region (IR).^31^, ^32^, ^33^ However, the innervation function of IR is spatially heterogeneous, that is, the density of nerve fibers, M receptors and *I*_K,Ach_ channel in the PLA and the junction of PV ostium and left atrium (PV‐LA) is greater than that in other left atrial regions; Tan et al also demonstrated that autonomic nerve density was highest in the LA within 5 mm of the PV‐LA junction by immunohistochemical staining on segments from normal human hearts.^34^ But the purpose of this innervation in normal hearts still remains unclear. Of these IRs, PLA is a representative region, where the content of vagus nerve fibers is seven times higher than sympathetic nerve fibers, suggesting the predominance of vagus nerve in PLA. The density of nerve fibers in PV‐LA junction is significantly higher than PV distal parts, and the sympathetic innervation appears to be superior. The regional differences in the conduction velocity and ERP of PLA and PV‐LA as a result of anatomical properties^31^, ^32^, ^33^ provide an important condition for the formation and spreading of driving rotor in atria. Using frequency‐domain analysis, Kalifa et al^35^ found that 80% of the maximal dominant frequency (DF_max_) sites of AF were located in the junction of PLA and PV, and that the rotor could stably anchor in this region, which was manifested by the DF_max_ sites with relatively high regularity index. Arora et al and our previous study^31^, ^32^ demonstrated that intervention of PLA with autonomic nerve blockers could extend the local ERP, lower atrial ERP dispersion and significantly reduce the induction rate and duration of AF.

**Figure 4:**
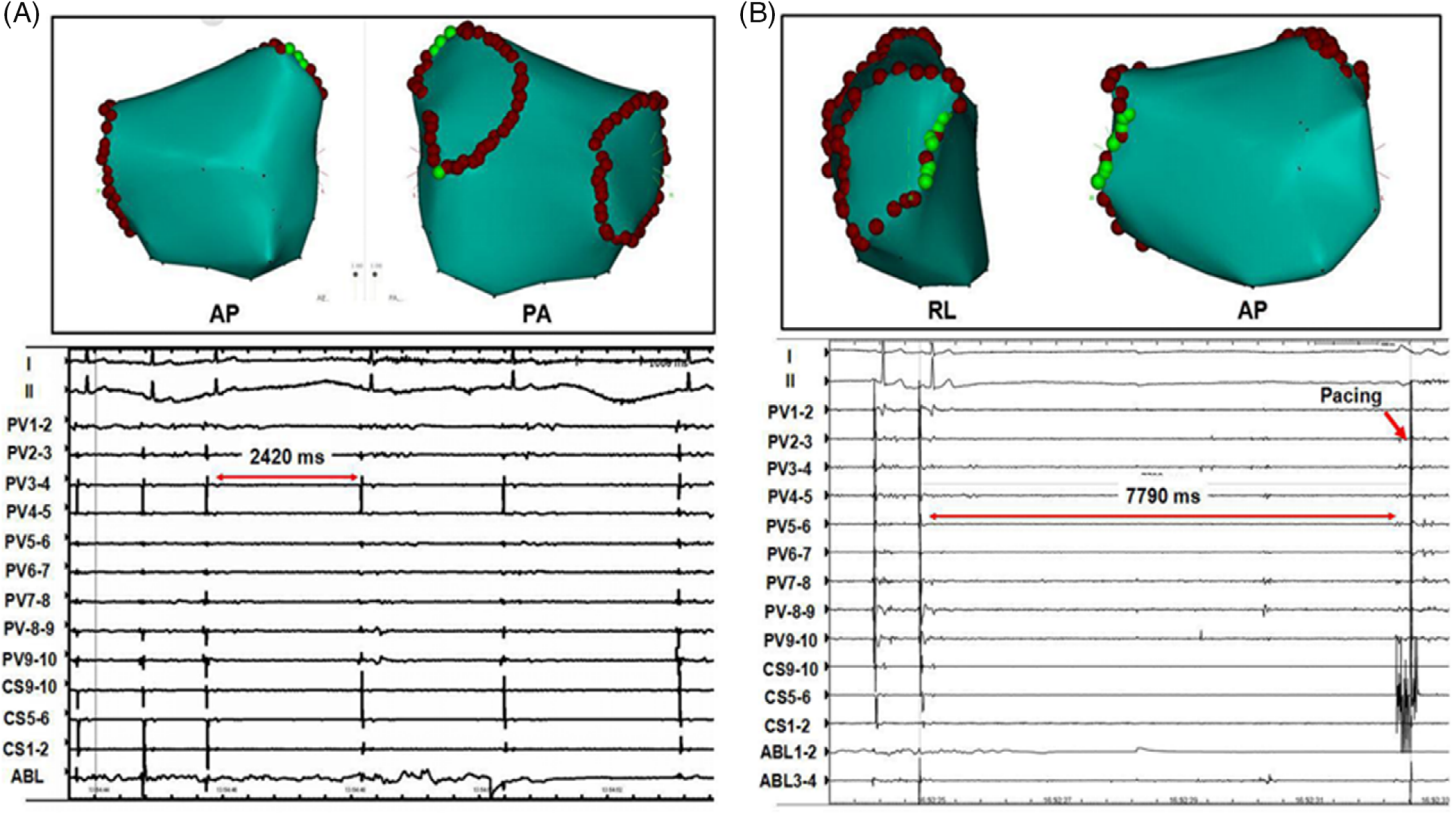
**A,** Left atrial electroanatomic maps in anteroposterior (AP) view and posteroanterior (PA) view, showing location of sites at which vagal response was elicited (green points) in ostia of left pulmonary vein. Application of radiofrequency energy at these sites induced transient bradycardia (RR interval = 2.4 seconds). **B,** Left atrial electroanatomic maps in right‐lateral (RL) view and anteroposterior view, showing location of sites at which vagal response was elicited (green points) in ostia of right pulmonary vein. Application of radiofrequency energy at these sites resulted in sinus pause (RR interval N 7.7 seconds), artificial pacing had to use at coronary sinus(red arrow). AP, anteroposterior view; PA, posteroanterior view; RL, right lateral view

Moreover, Chevalier et al^36^ provided a detailed description on the distribution characteristics of autonomic nerve at PV‐LA junction: for the longitudinal distribution, the density in the proximal segment of PV was greater than that in the distal segment, and the density of adrenergic and cholinergic nerves in the LA reached the peak within 5 mm of the PV‐LA junction and was higher than that in PV distal segment and LA proximal segment; for the transverse distribution, the density of two nerve fibers was higher in LSPV superior segment, RSPV anterior superior segment, LIPV and RIPV inferior segment. In consequence, the excitatory discharge of GP‐activated axons surrounding PV can reversely further activate GP and form a vicious cycle through the mutual activation between GP and axons, thereby leading to atrial remodeling and deteriorating to persistent AF. Katritsis et al^37^ recently conducted a multi‐center clinical randomized controlled study and reported that circumferential pulmonary vein isolation combined with denervation at PV ostium could improve the postoperative success rate of AF patients by approximately 30%. Notably, our study^38^ also confirmed that intervention of PV ostium (including: RSPV anterior wall and LSPV apex, etc.) with radiofrequency ablation can produce significant vagal reactions manifested by sinus arrest and atrioventricular conduction delay; modification of this region can significantly decrease the postoperative recurrence rate (Figure 4). These results fully demonstrate that there is an autonomic innervation region at PV ostium. This region, which is anatomically distant from GP but may have a cross‐linking relationship with GP, participates in the occurrence of paroxysmal AF as a CANS substrate. In conclusion, we believe that the IR region represented by PLA and PV‐LA junction is directly associated with CANS; the rapid electrical activity of atria in the IR region can reversely activate CANS and form a mutually‐promoting vicious cycle, thus, leading to atrial remodeling and further deteriorating to persistent AF; IR region may be an important part for the maintenance of paroxysmal AF.

## 4 THE RELATIONSHIP BETWEEN CANS AND COMPLEX FRACTIONAL ATRIAL ELECTROGRAMS

Although the original report by Nademanee et al^39^ shows that catheter ablation targeting complex fractional atrial electrograms (CFAEs) resulted in a 91% success rate in eliminating symptomatic AF, the subsequent clinical studies and meta‐analysis point out that CFAE ablation seems not to confer incremental benefit when performed in addition to PVI.^40^, ^41^In particular, the STAR‐AF II trial demonstrated that there were no reduction in the rate of recurrent AF when either CFAE ablation or linear ablation was performed in addition to PVI.^41^ Moreover, the mechanism(s) operative in the formation of CFAEs remains to be clarified, functional conduction block due to tissue anisotropy, colliding waveforms, migration rotors, and conduction slowing at critical parts of multiple re‐entrant circuits have been proposed as the underlying mechanisms.^35^, ^42^, ^43^ A striking finding in the report by Nademanee et al themselves is that most of the CFAEs were located at or around the sites where GPs were presumed to be.^39^ Several clinical studies have demonstrated that CFAEs were most commonly recorded at the PV‐atrial junction, where the tissue is richly innervated by autonomic neural elements.^44^, ^45^, ^46^ Recently, experimental studies on animal models have provided evidence that CFAEs induced by topical application of acetylcholine can be alleviated by ablating GP at a distance and can be eliminated when all the four major atrial GP were ablated, indicating that CFAEs may reflect the activity of the intrinsic cardiac ANS.^47^ Besides, the formation of CFAEs is accounted for by the rotor hypothesis. It states that fractionation of the electrograms occurs when the meandering rotor encounters heterogeneous substrate.^35^ As the rotors typically exhibit short and regular cycle length, the site showing maximal dominant frequency (DF_max_) through Fourier transformation has been proposed as a surrogate for the meandering rotor, and the outer limit of the DF_max_ domain is the area where the most propagation pattern variability and CFAEs activity occur. That is, CFAEs are the by‐product of the reentrant rotor that breaks down at its boundary. Moreover, to maintain a stable rotor, it required infusion of acetylcholine into the heart to keep the refractory period short and less heterogeneous, implying that the rotor hypothesis is also based on the autonomic activity of the CANS.

## 5 THE RELATIONSHIP BETWEEN CANS MODULATION AND AF

### 5.1 GP ablation in AF

A large number of previous basic studies have found that reducing ICANS activity through modifying GP with radiofrequency ablation can effectively decrease the induction rate of AF and inhibit atrial electrical remodeling.^26^, ^48^, ^49^, ^50^, ^51^, ^52^, ^53^ The target atrial GP mainly involve GP around the PV (left superior GP, left inferior GP, right anterior GP, and right inferior GP) (Figure 4). In addition, it has also been reported that the ablation of Marshall ligament GP and the third fat pad (aorta‐superior vena cava GP) can realize AF treatment as well.^26^, ^51^ However, GP ablation‐based CANS modification strategy failed to achieve the theoretical expectations for AF treatment in its clinical application. According to Mikhaylov, the 3‐year success rate of GP ablation in paroxysmal AF treatment was 34.3%.^52^ A recent multi‐center study^37^ showed that the 1‐year success rate of GP ablation alone in the treatment of paroxysmal AF was only 48%, while the success rate of pulmonary vein isolation (PVI) procedure could be enhanced by 18% in the case of combination with GP ablation.

In addition, there are some potential negative effects of GP ablation that we should concern. Osman et al. reported a case of a patient having GP ablation, who subsequently developed ventricular fibrillation after programmed ventricular stimulation.^54^ Mao et al determined a similar experimental result that epicardial GP ablation without additional atrial ablation increased the inducibility of atrial tachyarrhythmias, probably by decreasing the ERP of the atrial myocardium.^53^ The AFACT trial demonstrated that in patients with enlarged LAs, epicardial GP ablation during thoracoscopic surgery did not reduce AF recurrence, but was associated with more bleeding and significantly more pacemaker implantations because of sinus node dysfunction and atrioventricular block.^55^ However, we need to understand that the AFACT trial was a study on epicardial ablation, which could cause more lesion than endocardial ablation. The ablation extent may be associated with the prognosis, but current technology has made it impossible to either measure or quantify the depth of ablation.

**Figure 5:**
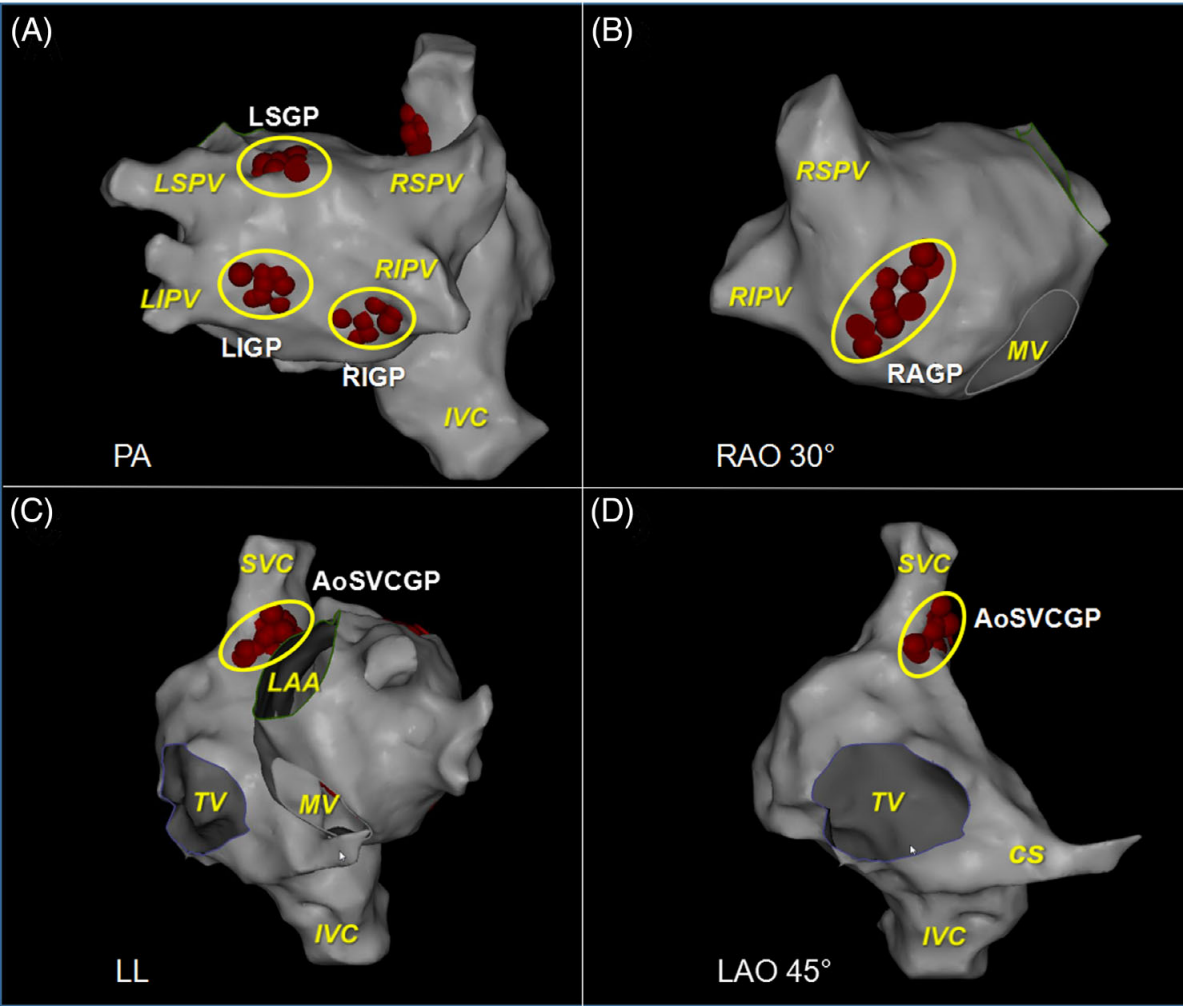
The location of ganglionated plexuses (GPs) in atrial electro‐anatomic maps. GPs sites are shown as red dots and yellow circles. **A,** LSGP, LIGP, RIGP distribute in the posterior left atrium near LSPV, LIPV, RIPV, respectively. **B,** RAGP distribute in the anterior left atrium near RSPV. **C, D,** Ao‐SVC GP located between the medial superior vena cava and aortic root. Ao‐SVC GP, aorta‐superior vena cava GP; CS, coronary sinus; IVC, inferior vena cava; LAA, left atrial appendage; LIGP, left inferior GP; LSGP, left superior GP; RAGP, right anterior GP; RIGP, right inferior GP; MV, mitral valve; SVC, superior vena cava; TV, tricuspid valve

Therefore, GP ablation can only be employed as a supplementary strategy for the paroxysmal AF PVI ablation at present. The potential reasons explaining the inconsistency between the clinical prognosis and basic research of GP ablation have been summarized as follows. First, the innervation region and interaction mechanism of GP remain unclear, and the research outcomes regarding denervation in the third fat pad, left atrial GP are still controversial. For example, Mao et al^53^ reported that left atrial denervation by GP ablation could increase the induction rate of AF. It is difficult to identify the actual damage range and extent of autonomic nerve after GP ablation, and the induction of CANS “re‐imbalance” by GP ablation requires to be further verified. Second, it is difficult to exactly locate GP. In current clinical trials, the sites of vagal reflex as located by high‐frequency stimulation may be just a result of axon and dendrite responses to the stimulation rather than where GP are located. Besides, the “anatomical” GP ablation is accompanied by certain blindness, and the individual differences in fat pad size and distribution cannot be avoided; more than one‐third of GP are located in the PLA and lie far from the fat pad,^33^ and the expansion of ablation area will increase the incidence of postoperative iatrogenic atrial tachycardia and flutter. Third, GP are located in the epicardial fat pad, it is hard to perform transmural lesion for complete autonomic denervation by the endocardial catheter ablation. Katritsis et al^37^ performed the PVI circumferential line in continuation with the GP ablation sites. Thus, during completion of the PVI, transmural lesions were delivered at the anatomic sites of GP. This ablation set undoubtly provide a evaluated criterion for transmural lesion and definitely delivered the lesion at the epicardial sites of GP, but the degree of fat pad damage remains unclear. Abstractly, the epicardial interventions may be the only way to achieve complete GP destroying. However, the AFACT trial^55^ seriously questions the role of epicardial fat pad/GP ablation in treating AF. Thus, there is lack of quantitative criteria for GP modification. Fourth, nerve regeneration and remodeling may occur after GP ablation. Sakamoto et al^56^ found that the vagal response was restored at 4 weeks after GP ablation and this reaction intensity was more significant than that before ablation. This finding suggests that the developed autonomic nerve regeneration and remodeling after GP ablation will result in changes in CANS functions. Furthermore, the regeneration speed and homogeneous distribution status of sympathetic and vagus nerve endings are also indefinite during this course. Therefore, the modification targeting to CANS substrate will remain an important and imperative issue in the future research of AF treatment (Figure 5).

### 5.2 Low‐level vagus nerve stimulation in AF

Previous study^57^ showed that low‐level vagus nerve stimulation (LL‐VNS, with a stimulus strength at 1 V below the threshold that immediately decelerate the sinus rate), was able to suppress stellate ganglion nerve activities and reduce the incidences of paroxysmal atrial tachyarrhythmias in ambulatory dogs, especially in the morning and decreased tyrosinehydroxylase positive cells in the left stellate ganglion 1 week after cessation of LLVNS. Other studies^57^, ^58^ also demonstrated that LL‐VNS at voltages substantially below the threshold for slowing the sinus rate or AV conduction, significantly increased the ERP in the atria as well as the PV myocardium, suppressed AF inducibility and decreased the duration of acetylcholine induced. In those experiments, LLVNS applied to both vagal trunks dissected in the neck. Based on the observation that transcutaneous electrical stimulation of the tragus, the anterior protuberance of the outer ear, where the auricular branch of the vagus nerve is located. This anti‐arrhythmic effect of LLTS was similar to those of LLVNS delivered to the cervical vagus nerve trunk(s). In a randomized clinical study,^59^ Stavrakis et al demonstrated for the first time in humans that the duration and inducibility of AF, as well as inflammatory cytokines were suppressed by low‐level transcutaneous electrical stimulation of the auricular branch of the vagus nerve. As a novel therapeutic approach, it takes advantage of the plasticity of the neural tissue to provide therapeutic advantage without damage to nerves or myocardium. As a non‐pharmacologic, non‐ablative neuromodulatory therapy for patients with paroxysmal AF, the long‐term safety, efficacy, and optimal stimulation protocol need further explored.

### 5.3 Renal sympathetic denervation in AF

Renal sympathetic denervation (RDN) has emerged as a novel approach for treatment of patients with resistant hypertension. Because sympathetic nerve activity is important in blood pressure control, reduction of sympathetic outflow may, in part, explain the reduction of blood pressure in some patients. Although the initial encouraging results of the open‐label simplicity HTN‐2 trial, the results of the larger blinded simplicity HTN‐3 trial were disappointing.^60^, ^61^ The same approach also used in controlling AF due to the mechanism of arrhythmia triggering through the reno‐cardiac axis. Little clinical evidence compared pulmonary vein isolation (PVI) alone vs PVI combined with RDN in AF patients.^62^ There was 69% of the patients who underwent the strategy of combined PVI and RDN were free of AF at 1‐year follow‐up compared to 29% in the PVI only group. But these AF patients have concomitant drug‐resistant hypertension demonstrated and RDN is associated with less recurrence of AF at intermediate‐term follow‐up in these patients. Thus, it is unclear whether the effect of RDN on AF recurrence was independent of the blood pressure control. Another study demonstrated that RDN has been used for ventricular rate control in AF and for reduction of AF episodes in patients with sleep apnea.^63^ But an experimental study of pig models revealed that RDN did not decrease the AF inducibility or atrial ERP, and it was of no effect in preventing atrial electrical remodeling during atrial fibrillation.^64^ However, there are some issues such as, the benefit of RDN in different types of AF (paroxysmal or persistent) and normotensive patients, remains unclear. Despite some ongoing clinical studies testing the hypothesis that concomitant renal denervation may improve the outcomes from catheter ablation of AF, there are still many vexing questions on the issue should be addressed by adequately powered well‐conducted randomized controlled trials.

## 6 CONCLUSION

Although the role of CANS in AF genesis has been demonstrated by a large number of basic and clinical studies, the CANS network and its regulating relationship of up and downstream remain unclear. Moreover, the therapeutic applications by autonomic modification, which base on CANS mechanism, still in “exploratory stage” and has not been applied extensively. Further studies are warranted to demonstrate these issues on the molecular, cellular, and tissue level, and confirm these novel therapeutic approaches in patients with AF.

## Supporting information


**Table S1.** Main experimental studies referred in part 1.1
**Table S2.** Main experimental studies referred in part 2.1
