# Supplementary material for: The cardiac autonomic nervous system: A target for modulation of atrial fibrillation
Source: Clin Cardiol. 2019 May 6;42(6):644–52. doi: 10.1002/clc.23190 (PMC6553352; doi:10.1002/clc.23190)
Supplement: Supplementary file 1 — Table S1. Main experimental studies referred in part 1.1 Table S2. Main experimental studies referred in part 2.1 [file CLC-42-644-s001.docx]

**Supplemental Table 1.** Main experimental studies referred in part 1.1

| Authors | Publication year | Animal models | Interventions | Outcomes/conclusions |
| --- | --- | --- | --- | --- |
| Lu ZB, Sunny S. Po et al. | 2009 | Mongrel dogs (acute AF model) | Stimulation of canine cervical vagal trunk with high-frequency current | **Stimulation of cervical vagal trunk**:  AF ERP ↓  AF incidence ↑  AF duration ↑  Atrial WOV ↑ |
| Lu ZB, Sunny S. Po et al. | 2008 | Mongrel dogs (acute AF model) | Drug blockage of ANS or GP ablation via the epicardium | **ANS blockage**：  Atrial heterogeneity ↓  Atrial electrical remodeling ↓ |
| Yeh YH, Nattel S et al. | 2007 | Mongrel dogs (chronic AF model) | Continuous atrial pacing for 4~6 weeks | **Persistent AF**:  Heterogeneity in the distribution and activity of atrial autonomic nerve ↑  M receptor quantity ↓  I_K,Ach_ channel density↓ |
| Jason Ng, Rishi Arora et al. | 2011 | Hound dogs (CHF model) | 1. Rapid ventricular pacing for 3 weeks  2.Sympathetic blockade  3.Parasympathetic blockade | 1.**CHF**:  nerve bundle size ↑  parasympathetic fibers/bundle ↑sympathetic fibrils density ↑  2. **Sympathetic blockade**:  AF dominant frequency ↓  3. **Parasympathetic blockade**:  AF duration ↓ |

AF=atrial fibrillation; ERP=effective refractory period; WOV=window of vulnerability; ANS=autonomic nerve system; GP= ganglionated plexi; CHF=chronic heart failure.

↑: increase after interventions; ↓: decrease after interventions.

**Supplemental Table 2.** Main experimental studies referred in part 2.1

| Authors | Publication year | Animal models | Interventions | Outcomes/conclusions |
| --- | --- | --- | --- | --- |
| Tan AY, Chen PS et al. | 2008 | Mongrel dogs (paroxysmal AF or AT model) | 1.Intermittent atrial pacing for 3±1 weeks  2.Extrinsic autonomic nerve activity recording  3.Cryoablation of sympathovagal nerves | 1.**Chronic AF or AT**  2.Simultaneous sympathovagal discharges immediately preceded 73% of the AF or AT onset  3.**Cryoablation of ANS**:  AF or AT eliminated, suggesting the causal relationship between ANS and AF or AT |
| Choi EK, Chen PS et al. | 2010 | Mongrel dogs (paroxysmal AF model) | Simultaneous recording of extrinsic and intrinsic autonomic nerve activities | ICANS activation preceded the onset of paroxysmal AF in nearly 100% cases, where 20% suffered the absence of ECANS activation, suggesting ICANS can trigger AF independent of ECANS |
| Lo LW, Sunny S. Po et al. | 2013 | Mongrel dogs after a right thoracotomy | SVC-Ao GP ablation | **Acute effects**:  ERP ↑  **Chronic effects**:  regional ERPs ↓  AF/AT burden ↑ |
| Emmanuel Moss, Pierre Page et al. | 2013 | Mongrel dogs | Left atrial GP stimulation | **Left atrial GP stimulation**:  LA and RA ERP changes  Bachmann conduction ↓  Sinuatrial dysfunction |
| Hou YL, Sunny S. Po et al. | 2007 | Mongrel dogs | 1.vagosympathetic trunk stimulation  2.RAGP ablation  3.LSGP ablation | 1.**vagosympathetic trunk stimulation**:  ERP ↓  AF inducibility ↑  2.**RAGP ablation**:  ERP shortening ↓  AF inducibility ↓  3.**LSGP ablation**:  ERP shortening ↓ |

AF=atrial fibrillation; AT=atrial tachycardia; ERP=effective refractory period; LA=left atrium; RA=right atrium; RAGP= right anterior ganglionated plexi; LSGP=left superior ganglionated plexi.

↑: increase after interventions; ↓: decrease after interventions.
